# Accurate V2X traffic prediction with deep learning architectures

**DOI:** 10.3389/frai.2025.1565287

**Published:** 2025-03-18

**Authors:** Ali R. Abdellah, Ahmed Abdelmoaty, Abdelhamied A. Ateya, Ahmed A. Abd El-Latif, Ammar Muthanna, Andrey Koucheryavy

**Affiliations:** ^1^Electrical Engineering Department, Faculty of Engineering, Al-Azhar University, Qena, Egypt; ^2^EIAS Data Science Lab, College of Computer and Information Sciences, Prince Sultan University, Riyadh, Saudi Arabia; ^3^Department of Electronics and Communications Engineering, Zagazig University, Zagazig, Egypt; ^4^Department of Mathematics and Computer Science, Faculty of Science, Menoufia University, Shebin El-Koom, Egypt; ^5^The Bonch-Bruevich Saint-Petersburg State University of Telecommunications, St. Petersburg, Russia; ^6^Applied Probability and Informatics, 'Peoples' Friendship University of Russia (RUDN University), Moscow, Russia

**Keywords:** 5G and beyond, V2X, AI, deep learning, BiLSTM, LSTM, GRU

## Abstract

Vehicle-to-Everything (V2X) communication promises to revolutionize road safety and efficiency. However, challenges in data sharing and network reliability impede its full realization. This paper addresses these challenges by proposing a novel Deep Learning (DL) approach for traffic prediction in V2X environments. We employ Bidirectional Long Short-Term Memory (BiLSTM) networks and compare their performance against other prominent DL architectures, including unidirectional LSTM and Gated Recurrent Unit (GRU). Our findings demonstrate that the BiLSTM model exhibits superior accuracy in predicting traffic patterns. This enhanced prediction capability enables more efficient resource allocation, improved network performance, and enhanced safety for all road users, reducing fuel consumption, decreased emissions, and a more sustainable transportation system.

## 1 Introduction

The rapid advancements in cellular networks, particularly 5G and beyond, offer transformative opportunities for emerging technologies like autonomous vehicles. These networks leverage Artificial Intelligence (AI) to optimize protocols and resource management, ensuring high-quality network performance with low latency and increased reliability. Such capabilities are essential for addressing autonomous vehicles’ stringent Quality of Service (QoS) requirements (Christopoulou et al., 2023; Sarker, 2022; Jiang et al., 2022; Ahmed et al., 2023). Driverless vehicles rely on continuous sensor data for real-time decision-making and complex manoeuvrers, particularly at high speeds. Beyond onboard processing, data sharing among nearby vehicles and infrastructure significantly enhances route optimization and long-term planning (Ali et al., 2024). This need for reliable connectivity has driven the development of advanced wireless networks enabling Vehicle-to-Everything (V2X) communication. V2X serves as a cornerstone for safe and efficient autonomous transportation, facilitating seamless communication among vehicles, pedestrians, and roadways while meeting the critical demands of modern mobility.

V2X communication protocols are revolutionizing transportation by enabling vehicles to connect seamlessly with each other, infrastructure, pedestrians, and the cloud. This interconnected ecosystem fosters real-time data exchange, paving the way for a future of improved traffic flow, enhanced safety, and reduced environmental impact. However, the burgeoning growth of V2X communication and overall traffic volume presents significant challenges. Increased congestion leads to delays, hinders economic development, and raises fuel costs, air pollution, and public health concerns. While intelligent traffic control methods hold promise, most are still in the early development stages. Therefore, a thorough examination of traffic control systems is necessary to pave the way for testing a new V2X-based smart traffic management theory designed to address congestion on our roadways (Li et al., 2023).

The advent of 5G wireless networks has introduced new challenges in network management and resource allocation due to increased complexity and data demands. Researchers increasingly use AI and big data techniques to address these challenges. Big data analytics provide valuable insights into the inner workings of 5G networks, facilitating a deeper understanding of network performance (Nair and Tanwar, 2023; Alhammadi et al., 2024). Machine Learning (ML), as a key component of AI, plays a vital role in tackling issues like network optimization and anomaly detection (Brik et al., 2022).

Deep learning (DL) methods have emerged within ML as powerful tools for predicting network traffic and performance. DL algorithms based on Neural Networks (NNs) have shown remarkable promise, particularly for processing historical data like traffic patterns to make accurate predictions (Sepasgozar and Pierre, 2022). Among DL methods, Recurrent Neural Networks (RNNs) are well-suited for handling time-series data, making them ideal for forecasting traffic patterns in 5G networks. Studies have demonstrated their effectiveness in predicting traffic congestion evolution (Abdellah et al., 2021; Tsourdinis et al., 2022).

Traditional RNNs, particularly unidirectional Long Short-Term Memory (LSTM) and Gated Recurrent Unit (GRU) networks, struggle to accurately model the complex, bidirectional temporal dependencies inherent in dynamic Vehicle-to-Everything (V2X) traffic patterns. These models, while applied to traffic prediction, lack the ability to simultaneously leverage information from both past and future time steps. To address this, we propose utilizing Bidirectional Long Short-Term Memory (BiLSTM) networks, which offer a significant advancement over unidirectional RNNs like LSTMs and GRUs for V2X traffic prediction (Gao et al., 2022). However, it is crucial to acknowledge their inherent limitations. The efficacy of BiLSTMs heavily relies on the availability of extensive, high-quality training data, posing a significant challenge in the inherently dynamic and often sparse V2X environment where data distribution can shift rapidly. Data dependency is exacerbated by the need for diverse sensor inputs, and the model’s performance may degrade substantially in scenarios with incomplete or noisy data, which are common in real-world deployments. Secondly, the computational efficiency of BiLSTMs demands careful consideration. Processing data in both forward and backward directions inherently increase computational load, potentially hindering real-time performance, particularly with growing data volume and network complexity. The associated latency could be a critical bottleneck in time-sensitive V2X applications.

Moreover, scalability remains a concern, as the model’s complexity might limit its applicability to large-scale deployments with numerous connected vehicles and infrastructure elements. Finally, real-world deployment challenges extend beyond computational aspects. Integrating BiLSTMs into existing V2X infrastructure necessitates seamless interoperability with diverse hardware and software systems, requiring significant standardization and validation. The model’s robustness against unexpected traffic anomalies, sensor failures, and network disruptions is paramount, demanding rigorous testing and fault-tolerance mechanisms. Furthermore, handling the inherent diversity of sensor data, including varying sampling rates and data formats, presents a significant engineering hurdle. These limitations underscore the necessity for robust evaluation methodologies and practical deployment strategies to ensure the reliable and effective utilization of BiLSTMs in V2X traffic prediction.

### 1.1 Main contributions and features of the proposed work

- We introduce a state-of-the-art time series forecasting model for ITS, which integrates cutting-edge DL models, including a novel Bidirectional Long Short-Term Memory (BiLSTM) architecture, achieving superior traffic pattern prediction accuracy over traditional approaches.
- We provide comparative analysis against LSTM and Gated Recurrent Unit (GRU) models, demonstrating the effectiveness of our architecture in capturing complex traffic dynamics in V2X networks.
- Our proposed architecture has significant implications for developing more efficient and effective traffic management systems and provides a foundation for future research.
- The final simulation run yielded the following key findings:
- Superior Performance of BiLSTM: Our research reveals that the BiLSTM architecture achieves the highest prediction accuracy among the compared models, demonstrating its effectiveness in traffic flow prediction within V2X networks.
- Our research demonstrates that both BiLSTM and GRU models significantly outperform the traditional LSTM model in traffic flow prediction within V2X networks. BiLSTM achieves the highest accuracy, showcasing its effectiveness in capturing complex traffic dynamics.
- Efficiency Considerations: While the traditional GRU model exhibits the fastest processing time in most cases, the BiLSTM model offers a good balance between accuracy and processing efficiency. Notably, The LSTM model, however, exhibited lower prediction performance compared to the others.
- Interestingly, our experiments reveal a clear correlation between data transmission rate and DL model performance. For all three models (BiLSTM, GRU, and LSTM), the optimal performance was identified at 4 packets/s transmission rate. This finding suggests a potential trade-off between data freshness and computational efficiency.

This article explores the potential of DL for V2X communication. We review relevant literature in Section 2, providing context for using DL in this field. Section 3 introduces the concept of DL and its application in V2X communication—Section 4 details our proposed approach, followed by the findings of our work in Section 5. Finally, Section 6 concludes the paper by summarizing the key takeaways and outlining future directions.

## 2 Related work

Accurately predicting traffic patterns is crucial for optimizing wireless network performance. Traffic prediction enables proactive resource management, congestion control, and improved QoS provisioning. This review focuses on recent advancements in traffic prediction techniques for wireless networks, analyzing research trends with a particular emphasis on ML algorithms for traffic forecasting. By summarizing the strengths and limitations of existing approaches, we aim to identify opportunities for further research in this domain.

Before delving into the literature, it’s crucial to acknowledge the specific challenges of Intelligent Transportation System (ITS) datasets, particularly their often-limited size. This constraint significantly influences the choice of predictive models. We deployed LSTM, GRU, and BiLSTM for traffic prediction in ITS datasets due to their suitability for handling small-size data, a common challenge in this domain. As sub-RNN methods, LSTMs and their variants are particularly effective at capturing complex temporal patterns in small-size data, making them a natural choice for ITS datasets.

While various methodologies exist for traffic prediction, their applicability to small data ITS datasets is limited. For example, Graph Convolutional Networks (GCNs) (Khan et al., 2023) and Transformer-based models (Tariq et al., 2024), known for their success in capturing complex spatial and temporal dependencies, typically require substantial data to learn meaningful representations. Similarly, traditional statistical methods like ARIMA (Tran et al., 2022) and machine learning approaches like Support Vector Machines (SVM) (Wang et al., 2022), while valuable in other contexts, may struggle to capture the complex, nonlinear dynamics inherent in ITS data with limited training samples. This limitation highlights a critical gap: the need for robust prediction models that perform effectively with small-size ITS datasets.

Several studies have investigated the application of LSTM models for traffic prediction, highlighting their effectiveness and adaptability in diverse contexts. For instance, Khan et al. (2023) identifies challenges in short-term traffic forecasting with LSTMs and suggests areas for future research to overcome these limitations. Similarly, Tariq et al. (2024) leverages LSTM-based prediction to address network slice traffic demand challenges in 5G network slicing, emphasizing its potential for improving connectivity despite non-uniform slice deployment within registration areas. In a different study (Tran et al., 2022), researchers applied an LSTM model with optimized hyperparameters to predict traffic speeds on complex multi-path roadway systems in Vietnam, demonstrating its ability to capture traffic flow dynamics effectively in emerging country scenarios. Further extending the use of LSTM models, Wang et al. (2022) presented an approach for environmental movement prediction, focusing on enhancing security decision-making and local path planning through accurate movement pattern forecasts. Gao et al. (2022) refined the LSTM architecture to forecast unpredictable 5G traffic sequences. Their adaptive LSTM system dynamically adjusted the number of hidden layers and units, significantly improving prediction accuracy for mobile network traffic. These studies underscore the versatility and potential of LSTM models for tackling diverse traffic prediction challenges across different domains and contexts.

Abdellah et al. (2022) investigated the application of DL for forecasting energy consumption in drone-based mobile edge computing (MEC) systems. They employed an LSTM network, a type of RNN adept at handling sequential data like time series. Their approach evaluated the impact of the learning rate, a hyperparameter controlling the speed of model updates during training, on the prediction accuracy. The authors utilized RMSE and MAPE to assess the performance across four learning rates. This analysis aimed to identify the configuration that yielded the most accurate forecasts and the most significant overall improvement in prediction performance. In Wang et al. (2022), the author identified limitations in current mobile network traffic analysis, precisely its inability to capture the spatiotemporal dynamics of traffic congestion. They proposed a novel method that leverages time series similarity-based graph attention networks to address this. This approach combines the strengths of time series analysis, which excels at capturing temporal patterns, and graph theory, which excels at Modeling relationships between network elements, to provide a more comprehensive understanding of mobile network traffic patterns.

To address limitations in traditional handover methods for high-speed railways (e.g., delays), Lu et al. (2022) proposes an LSTM-based prediction method that can potentially reduce handover delays and enhance the overall user experience of mobile internet services for passengers. This contributes to the ongoing research on applying ML for handover prediction in high-speed mobility scenarios. Bondaruc et al. (2023) introduces an Edge Computing architecture tailored for Smart Parking. This architecture leverages edge devices with GRU-based DL models to analyze sensor data (e.g., ultrasonic or magnetic sensors) and predict real-time parking space availability. The processed data can be further aggregated and analyzed by cloud-based systems to provide broader insights into parking trends and optimize city-wide management strategies.

Alzaghir et al. (2022) explores RNNs, specifically NARX neural networks, for predicting time series energy consumption in UAV-based MEC. RNNs are a powerful AI technique that handles sequential data, making them well-suited for traffic prediction tasks. The paper evaluates the prediction accuracy using metrics like RMSE and MAPE. Zhao et al. (2023) introduced a Nonlinear Autoregressive (NAR) neural network for ultra-short-term electric vehicle charging load forecasting at charging stations. This approach leverages the NAR model’s ability to capture nonlinear and dynamic features, leading to improved prediction accuracy compared to the Backpropagation (BP) neural network. Nabi et al. (2023) developed a DL approach to improve LTE network management. This method, called the Fusion Model, forecasts multiple data streams (multivariate traffic forecasting) and optimizes radio settings (radio parameter optimization). Combining these techniques, the Fusion Model enhances overall network management and QoS in cellular networks.

Our research builds upon this foundation by specifically applying LSTM, GRU, and BiLSTM models to address the challenges of small-size ITS datasets. By evaluating and comparing these RNN variants, we aim to provide insights into their effectiveness in capturing the complex temporal patterns inherent in such data, thereby contributing to developing more robust and reliable traffic prediction systems for wireless networks.

While promising, these studies have limitations in ML approaches and accurately predicting wireless network traffic. A comparative study of existing network traffic prediction methods, as presented in Table 1, further underscores these challenges.

**Table 1:** Comparative study of existing network traffic prediction methods.

References	Research area	Technique	Key findings/contributions	Strengths	Limitations/challenges
Khan et al. (2023); Tariq et al. (2024); Tran et al. (2022); Wang et al. (2022); Gao (2022)	Wireless Network Traffic Prediction	LSTM	Effective for short-term traffic forecasting. Adaptable to diverse contexts (5G slicing, roadway systems, environmental movement).	High accuracy captures temporal dependencies.	Potential for overfitting, computational cost, and difficulty in tuning hyperparameters.
Abdellah et al. (2022)	Drone-based MEC Energy Consumption	LSTM	Evaluated the impact of learning rate on prediction accuracy.	Handles sequential data effectively.	Requires careful hyperparameter tuning (learning rate).
Wang et al. (2022)	Mobile Network Traffic Analysis	Time Series Similarity-based Graph Attention Networks	Captures spatiotemporal dynamics of traffic congestion.	Combines strengths of time series analysis and graph theory.	The complexity of implementation and training.
Lu et al. (2022)	High-Speed Railway Handover	LSTM	Reduces handover delays and improves user experience.	Potential to enhance mobile internet services in high-speed mobility scenarios.	Requires accurate and real-time data collection.
Bondaruc et al. (2023)	Smart Parking	GRU	Predicts real-time parking space availability.	Enables efficient parking management and resource allocation.	Dependence on accurate sensor data and robust edge computing infrastructure.
Alzaghir et al. (2022)	UAV-based MEC Energy Consumption	NARX Neural Networks	Predicts time series energy consumption.	Handles sequential data, suitable for traffic prediction.	Requires careful selection of network architecture and training data.
Zhao et al. (2023)	Electric Vehicle Charging Load Forecasting	NAR Neural Network	Capturing nonlinear and dynamic features improves prediction accuracy compared to the BP neural network.	High accuracy, handles nonlinear relationships.	May require significant computational resources for training.
Nabi et al. (2023)	LTE Network Management	Fusion Model (multivariate traffic forecasting)	Forecasts multiple data streams and optimizes radio settings.	Enhances network management and QoS.	Requires accurate data collection and processing of multiple data streams.
This paper	V2X Traffic Prediction	DL with BiLSTM	Superior accuracy in predicting compared to the baseline model	“Improved network efficiency and user safety	–

## 3 DL approach in V2X communications

In recent years, ITS has been studied in detail and developed rapidly (Oladimeji et al., 2023). ITS integrates information, communication, and transportation technologies to improve traffic management, safety, network efficiency, and public transport management. Long- and short-term traffic information significantly impacts the quality of ITS operations, especially in traffic management. The latter is a priority for private and commercial road users. Predicting traffic in real time is challenging due to its stochastic and nonlinear nature. Current solutions mainly use traditional ML models to estimate traffic flow. Although they can adapt to seasonal variations, these approaches cannot accurately represent the nonlinear and probabilistic aspects of the phenomenon (Moushi et al., 2023). Recent advances in traffic prediction have successfully been demonstrated using ML-based models like ARIMA (Ospina et al., 2023) and Kalman filters (Gyeera et al., 2023). More complicated algorithms are employed to enhance traffic prediction performance by including periodicity.

**Figure 1:**
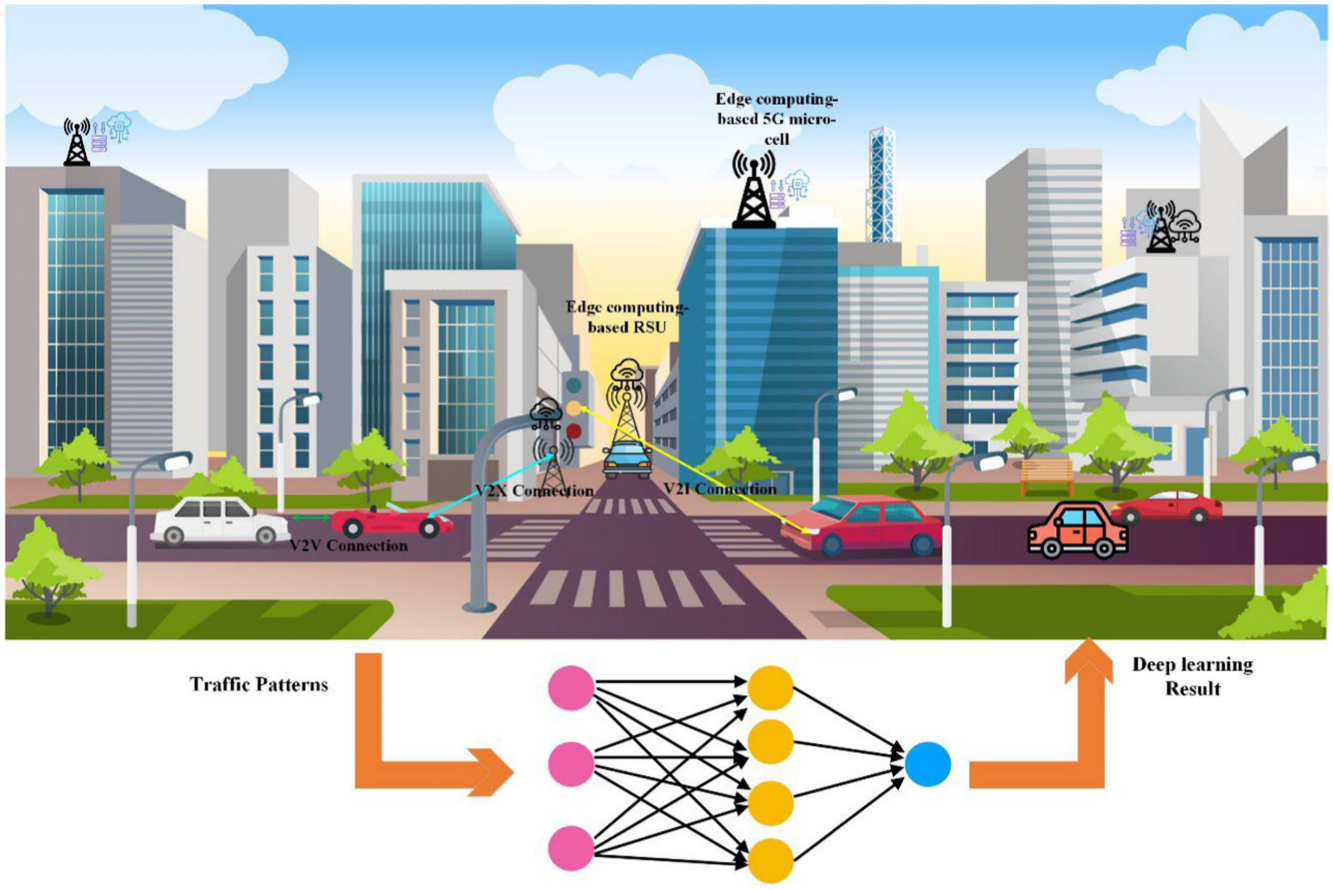
A V2X network with various communication types and interconnection with the DL model.

While advancements in AI and ML provide potent tools for traffic analysis, particularly with RNNs like BiLSTM, GRU, and LSTM, classical statistical methodologies remain crucial. Their importance extends beyond prediction, offering valuable insights into underlying traffic patterns (Abdellah and Koucheryavy, 2022). Data from real-world environments is essential for running ML models. Software traffic simulators can supplement accurate data when unavailable or insufficient, such as when they do not span the necessary time range. We compared the proposed BiLSTM predictor with the baseline LSTM and GRU predictors trained on actual data with data from the MATLAB simulator based on the Smart City traffic model for vehicle movements. Figure 1 shows V2X networks and the interconnection of cellular network infrastructure using DL-based models.

## 4 Proposed work

While numerous studies have leveraged ML techniques for early prediction of legal outcomes using historical data [e.g., NARX (Abdellah et al., 2021), ARIMA (Yan et al., 2022), XGBoost (Ban et al., 2022), LSTM (Yun et al., 2021; Mpinga et al., 2023; Kavehmadavani et al., 2023), Random Forest (Wang et al., 2022)], their focus has primarily been on procedural decisions. These approaches face limitations: (i) inadequate selection of factors influencing legal decisions and (ii) the inability of traditional encoders to capture complex relationships between those factors within legal data.

This study addresses the critical challenge of accurate traffic prediction in 5G and beyond mobile networks while ensuring QoS. Recognizing the limitations of existing approaches, we propose a novel framework that leverages DL architectures trained on V2X communication data, precisely the number of packets transmitted per second. This approach eliminates the need for prior knowledge of environmental conditions (e.g., channel statistics) and capitalizes on the powerful learning and generalization capabilities of DL.

Our proposed framework utilizes BiLSTM predictors, comparing their performance against baseline unidirectional LSTM and GRU architectures—variants of RNNs designed to address the vanishing gradient problem. We evaluate the prediction performance using RMSE, MAPE, and processing time metrics. The best-performing predictor will be selected to enhance network performance through improved QoS, optimized resource management, enhanced network security, and improved resolution of operational challenges.

All simulations and programming were conducted using MATLAB software. A simulated V2X system generated the training dataset. Before training, the collected V2X dataset underwent thorough assessment, cleaning, and preprocessing. The dataset was split into training (70%) and testing (30%) sets. Input data was normalized to the range [0, 1] for optimal training. The DL models (BiLSTM, GRU, and LSTM) were trained using the Adam optimizer, with Mean Squared Error (MSE) as the loss function, 1,000 epochs, a 0.01 learning rate, and a batch size 32.

During training, the network adjusts its weights using the delta rule to minimize errors between predicted and observed outputs. After fitting the training models, network weights and biases were modified to obtain the gradient of the loss function. This process was iteratively repeated to minimize output error. The performance of the trained models was subsequently evaluated on the held-out test set.

**Figure 2:**
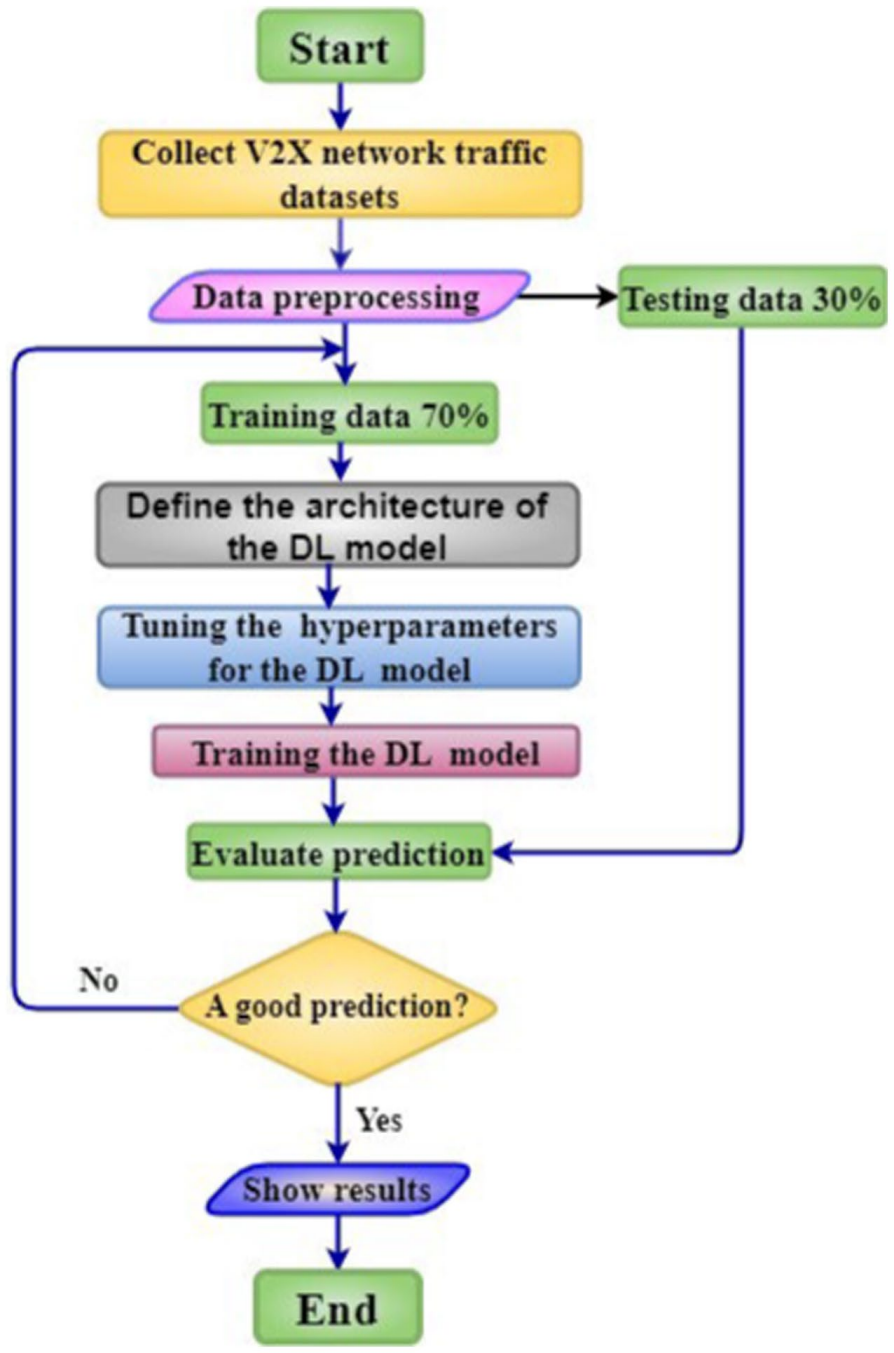
Flowchart for the proposed work.

To evaluate the effectiveness of our proposed models, we compared them based on accuracy (RMSE and MAPE) and efficiency (processing time). In traffic prediction, where the target variable is continuous (e.g., traffic flow, speed), the primary objective is to minimize the difference between predicted and actual values. RMSE quantifies this difference in the relevant units, providing a direct measure of prediction error. Monitoring RMSE during training helps assess model learning progress and identify areas for improvement. We can evaluate model performance without a separate validation set by splitting the data into training and testing sets. The RMSE calculated on the test set reflects the model’s ability to generalize to unseen data, making it a crucial indicator for real-world applicability. A flowchart summarizing our proposed work is presented in Figure 2.

### 4.1 LSTM predictor

LSTM models are powerful tools for processing sequential data. Unlike standard RNNs, which struggle with long-term dependencies due to the vanishing gradient problem, LSTMs excel at remembering and utilizing information over extended periods. This capability stems from their core architecture, which includes selective memory cells and three critical gates: forget, input, and output.

The selective memory cell is a component of the LSTM unit that functions as a gate of storage, allowing for the selective retention or discarding of information. Forget gate (*f_t_*) acts as a filter, deciding what information to keep from the previous cell state (*c_t−1_*). It analyses the current input (*x_t_*) and the prior output (*h_t−1_*) to determine which parts of *c_t−1_* are still relevant. Unnecessary information is selectively discarded, ensuring the memory cell is not overloaded with irrelevant details. While the forget gate focuses on removing the old, the input gate (*i_t_*) directs the flow of new information. It considers the current input (*x_t_*) and the previous output (*h_t−1_*) to create a candidate for the updated cell state (*c_t_*). The Candidate Cell State (*c’_t_*) is a vector formed by merging *x_t_* and *h_t−1_* using a *tanh* activation function to create the *c_t_* vector. It indicates the new information that can be stored based on the current conditions. Finally, the output gate (*o_t_*) controls what information from the current cell state (*c_t_*) is ultimately exposed as the hidden layer output (*h_t_*). It examines the internal state (*c_t_*) and the previous output (*h_t−1_*) to determine the most valuable information to share with the network.

**Figure 3:**
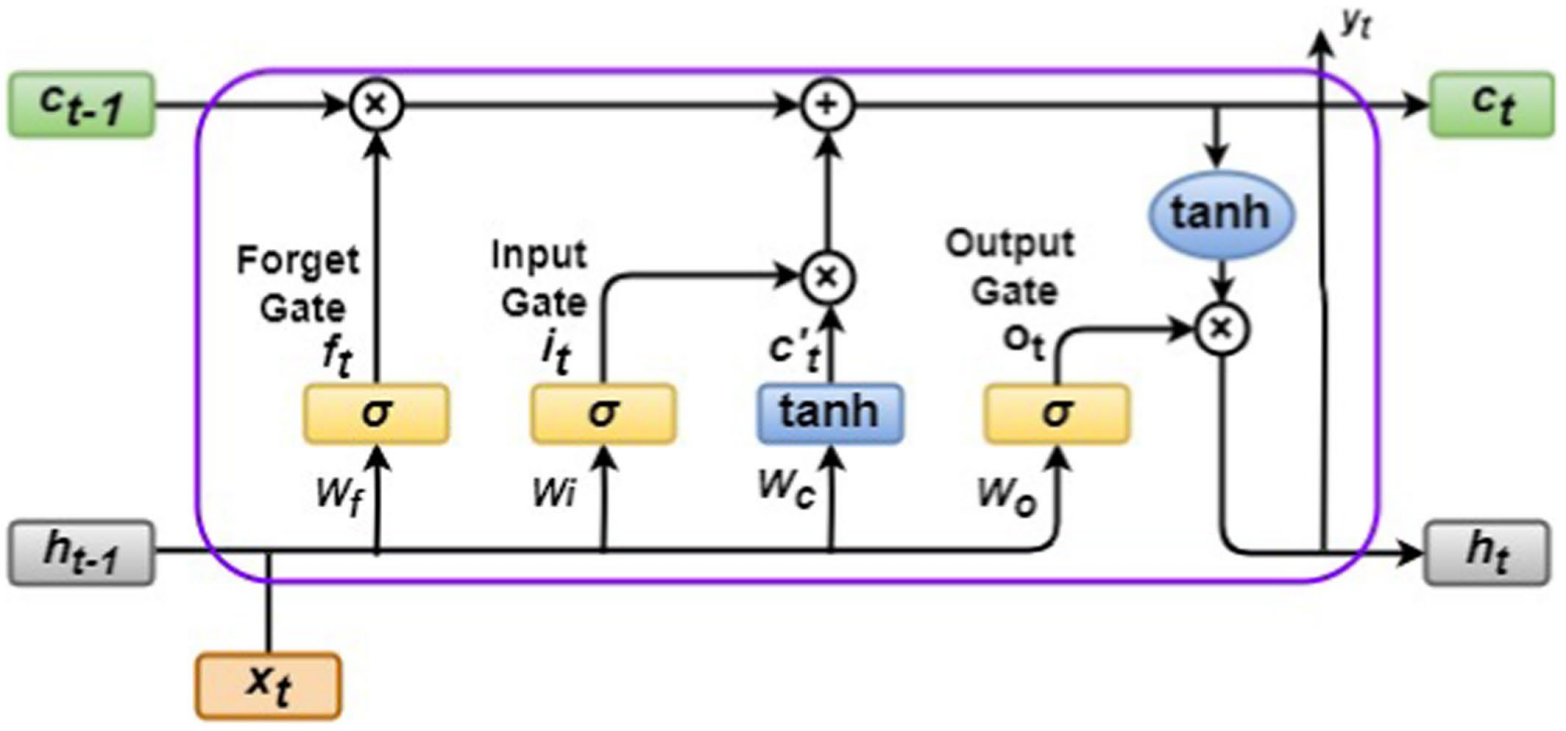
LSTM architecture.

By effectively managing information flow through these gates, LSTMs can learn long-term dependencies within sequential data and address the vanishing gradient problem that plagues traditional RNNs. The gates allow the network to selectively remember and forget information across long sequences, making them a powerful tool for various applications like machine translation, speech recognition, and time series forecasting. The LSTM architecture is shown in Figure 3, and its equations are listed below in Equations 1–6:


(1) $$i_{i}=\sigma \;\left(w_{i}\;x_{t}+u_{i}\;h_{t-1}+\mathrm{bi}\right)\text{,}$$

(2) $$f_{t}=\sigma \;\left(w_{f}\;x_{t}+u_{f}\;h_{t-1}+bf\right)$$

(3) $$o_{t}=\sigma \;\left(w_{o}\;x_{t}+u_{o}\;h_{t-1}+\mathrm{bo}\right)$$

(4) $${c^{’}}_{t}=\tanh\;\left(wc\;xt+uc\;h_{t-1}+bc\right)$$

(5) $$c_{t}=f_{t}⊙c_{t-1}+i_{t}c\prime_{t}$$

(6) $$h_{t}=o_{t}⊙\tanh\left(c_{t}\right)$$

The sigmoidal function is denoted by σ. The input vectors are represented by *x_t_*, the hidden state by *h_t_,* the weight vectors by *w* and *u,* and the bias vectors are denoted by *b*. The operator ⊙ denotes the Hadamard (element-wise product). The subscript *t* indexes the time step.

### 4.2 GRU predictor

GRU (Reddy and Parvathy, 2022; Wang et al., 2022), a type of RNN, outperforms LSTM in some cases. LSTM is more accurate when processing longer sequences, whereas GRU is more rapid and needs less memory. Furthermore, GRUs address the issue that vanilla RNNs face with diminishing gradients (values utilized for adjusting network weights). As the gradient propagates backward, it can become too small to affect learning over time, making the NN untrainable. When a layer in NN cannot train, RNNs can successfully “forget” extended sequences.

**Figure 4:**
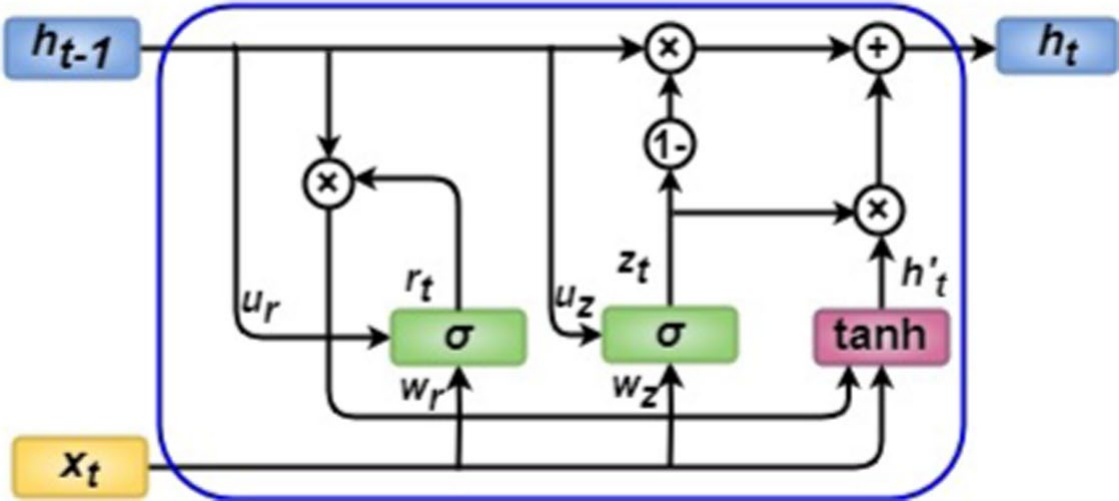
GRU architecture.

GRUs handle this issue through two gates: update and reset, as shown in Figure 4. The gates can be trained to remember previous information and determine which data should be output. This allows relevant information to be transmitted in a sequence of events to make better predictions. LSTMs and GRUs are comparable, although GRUs contain fewer parameters. Like LSTMs, they have gated units that control data transmission within the unit but have no memory cells. GRUs do not have an output gate like LSTMs, so their contents are visible. Equations 7–10 can be used to formulate the GRU. Initially, for *t_0_ = 0*, the output vector is *h_0_* = 0.


(7) $$r_{t}=\sigma \left(W_{r}x_{t}+U_{r}h_{t-1}+b_{r}\right)$$

(8) $$z_{t}=\sigma \left(W_{z}x_{t}+U_{z}h_{t-1}+b_{z}\right)$$

(9) $${\tilde{h}}_{t}=\tanh\left(W_{h}x_{t}+U_{h}\left(r_{t}⊙h_{t-1}\right)+b_{h}\right)$$

(10) $$h_{t}=\left(1-z_{t}\right)⊙h_{t-1}+z_{t}⊙{\tilde{h}}_{t}$$

The reset gate is denoted by $r_{t}$ , the update gate by $z_{t}$ , the input vector by $x_{t}$ , and the output vector by $h_{t}$ . Like LSTMs, *W* denotes the weight vector, *b* denotes the bias vector, *σ* for sigmoidal activation, and tanh denotes the hyperbolic tangent activation function.

### 4.3 BiLSTM predictor

BiLSTM (Wen et al., 2023; Hussain et al., 2023) is RNN, unlike standard LSTM, that develops long-term bidirectional connections between time steps in a time sequence or data series. These connections allow the RNN to learn from the entire series at every time step. BiLSTM adds a layer to reverse information flow. The additional LSTM layer reverses the input sequence. We average, add, multiply, or connect the outputs from both LSTM layers. BiLSTM integrates two hidden LSTM layers with different directions to the same output to overcome the disadvantage of a single LSTM cell that can only receive inputs from the past but cannot use future data. With this structure, the output layer can use related information from past and future situations.

**Figure 5:**
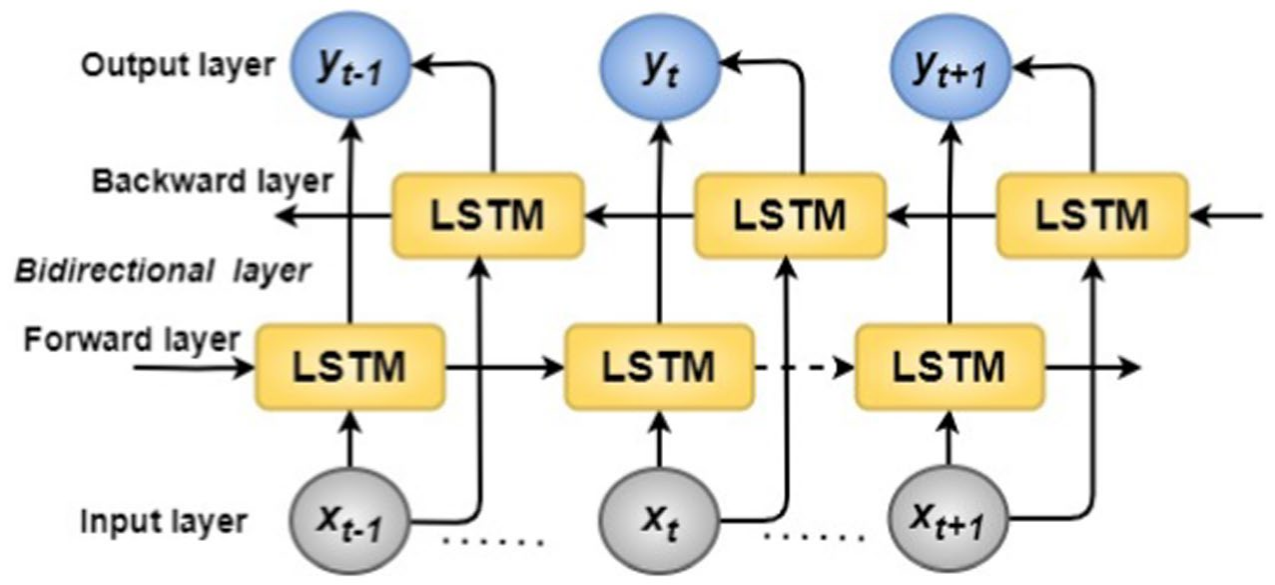
Bidirectional LSTM architecture.

BiLSTM network processes the input sequence (*x_t_*) in both forward and backward directions. We can denote the hidden states of the forward and backward directions at time step t for the i^th^ BiLSTM layer as $h_{f\left(t\right)}$ and $h_{b\left(t\right)}$ , respectively. The final hidden state (*h_t_*) in BiLSTM captures information from both directions by combining the $h_{f\left(t\right)}$ and $h_{b\left(t\right)}$ . The final output (*y_t_*) of the BiLSTM network is typically obtained by applying a nonlinear activation function (often tanh or sigmoid) to *h_t_*. Figure 5 illustrates the BiLSTM architecture. Figure 5 depicts the BiLSTM architecture. The final output of BiLSTM can be outlined by the following Equations 11–14:


(11) $$h_{f\left(t\right)}=\mathrm{LSTM}\left(x_{t},h_{f\left(t-1\right)}\right)$$

(12) $$h_{b\left(t\right)}=\mathrm{LSTM}\left(x_{t},h_{b\left(t+1\right)}\right)$$

(13) $$h_{t}=\left[h_{f\left(t\right)};h_{b\left(t\right)}\right]=h_{f\left(t\right)}⊙h_{b\left(t\right)}$$

(14) $$y_{t}=\sigma \left(W_{ht}*h_{t}+b_{h}\right)$$

### 4.4 DL dataset collection and preprocessing

**Figure 6:**
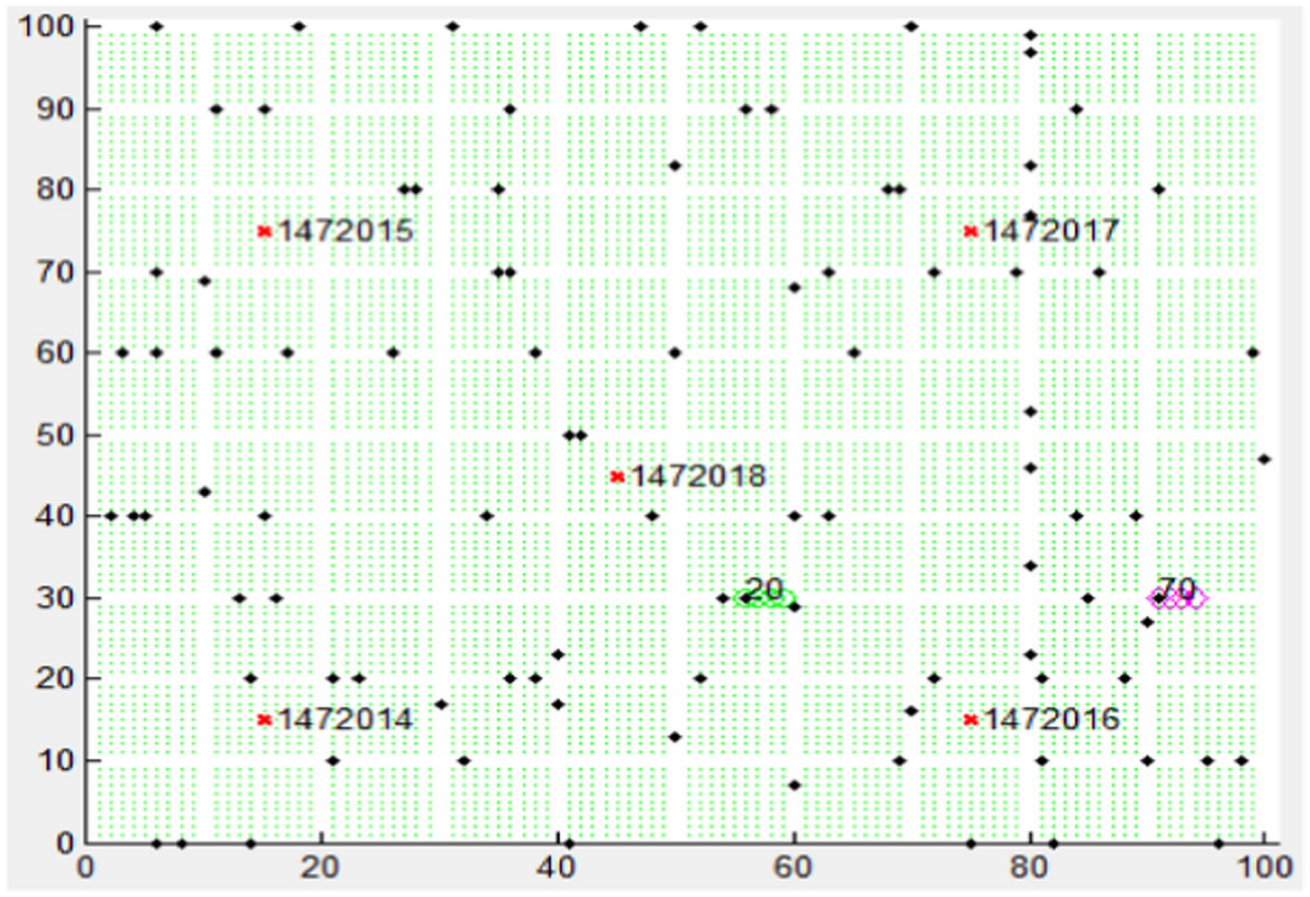
V2X network simulation: node movement on desired routes.

This work used a simulated V2X system dataset to train a DL model. All simulations and programming were conducted using MATLAB software. Figure 6 depicts the simulated V2X system, representing an intelligent transportation system within a smart city.

The simulated city environment encompasses a 100 × 100 m unit area on the x and y axes. A mobility model was implemented to define fixed paths for city border nodes, allowing movement in any direction. The nodes depicted as dots in the figure represent Roadside Units (RSUs) along with their corresponding positions determined by the network structure and configuration numbers. The simulation source node is at 20, and the destination node is at 70. The simulation module plays a crucial role in visualizing the network architecture and establishing start and end points for the simulation. An exciting aspect of this simulation is the ability for randomly moving nodes to connect with distant nodes due to the strategic placement of RSUs within the simulated map. This functionality facilitates communication between RSUs and moving vehicles, enabling the transmission of traffic information and critical safety alerts. In a simulated V2X, the road network consists of a 10×10 grid layout with horizontal and vertical lanes. Each road segment can have a specific number of lanes.

This research investigates throughput prediction, a crucial factor for QoS assurance in V2X communications. We utilize a dataset comprising 130 time-series samples of throughput values generated by network flow rules. These throughput values are the primary input for training our ML model.

High-quality data is essential for accurate time series forecasting. Time series forecasting scenarios require that the data be organized in a specific way, considering the data quality, depending on the model used for data preparation. Data cleaning and preprocessing were used to ensure the data’s integrity by identifying and removing errors, inconsistencies, and outliers. Missing values are addressed using appropriate techniques, such as imputation or deletion. Feature engineering, transforming the data into a format suitable for the chosen predictive model. This can include methods such as scaling or normalization, where all features are brought into a specific range (0 to 1).

The prepared data is fed into the ML model for training. This process involves two main steps: defining the model architecture and training the model itself. First, the network architecture is specified. This includes choosing the number of layers, the number of neurons within each layer, and the activation functions that determine how the model processes information. Second, the training process is configured. This involves selecting an optimizer (like Adam) to guide the model towards minimizing the difference between predicted and actual values. We also set training parameters like the learning rate, which controls how much the model adjusts its weight with each iteration, and the number of training epochs, which determines how often the model sees the entire training data set. Finally, a minimum acceptable error threshold is established for the predictions. Once trained, the model’s generalizability is evaluated using unseen data. This involves testing the model’s performance on data it has not encountered before. Predicted values are compared to actual values to assess accuracy. If the results are satisfactory, the predicted values undergo post-processing to determine the final expected output.

## 5 Simulation results

This study will train DL models to precisely predict traffic flow in a V2X environment at 4, 6, 8, 12, and 14 packets/s packet rates. Model performance will be evaluated using RMSE, MAPE, and processing time. These metrics are standard for comparing traffic stream forecasting models, allowing for direct comparison with existing methodologies. Lower RMSE and MAPE values indicate superior prediction accuracy, while processing time reflects computational efficiency. The formulas for RMSE and MAPE are presented below:


(15) $$\mathrm{RMSE}=\sqrt{\frac{1}{N}\overset{n}{\underset{i=1}{\sum }}{\left(y_{i}-{\hat{y}}_{i}\right)}^{2}}$$

(16) $$\mathrm{MAPE}=\frac{1}{N}\overset{n}{\underset{i=1}{\sum }}|\frac{y_{i}-{\hat{y}}_{i}}{x_{t}}|$$

Where *N* is the total number of data points considered, $y_{i}$ denotes the actual value, while ${\hat{y}}_{i}$ Denotes the predicted value.

To significantly enhance our prediction capabilities, we prioritize QoS demands, implement robust resource management, strengthen security measures, and proactively address other operational challenges. This is achieved through the deployment of an ML model. The model was trained in over 1,000 epochs with 200 hidden neurons, utilizing a batch size of 32, a learning rate of 0.01, and the Adam optimizer. The simulation parameters for DL models are provided in Table 2.

**Table 2:** DL model simulation parameters.

Parameter	Value
Simulation tool	MATLAB R2022b
DL models	LSTM/BiLSTM/ GRU
Number of layers	Single layer of (LSTM/BiLSTM/ GRU)
Hidden neurons	200
Optimization algorithm	Adam Optimizer
Loss function	MSE
State activation function	Tanh
Gate activation function	Sigmoid
Initial learning rate	0.01
Learn rate drop factor	0.2
Dropout	0.5
Gradient threshold	1
Epoch	500
Batch size	32
Input feature	V2X Throughput
Input dimension	130 samples (130 × 1)
Learning rate	0.01
Data split	70% Training, 30% Testing
Normalization	[0,1]
Performance metrics	RMSE/MAPE

Table 3 summarizes the performance of V2X traffic forecasting models across different transmission rates. It evaluates three key metrics: RMSE, MAPE, and processing time. The table considers transmission rates ranging from 4 to 14 packets/s.

**Table 3:** V2X traffic predictions summary using RMSE, MAPE, and the processing time.

Data rates (packets/s)	LSTM			BiLSTM			GRU		
	MAPE	RMSE	Processing time	MAPE	RMSE	Processing time	MAPE	RMSE	Processing time
4	10	0.5427	78	**8**	**0.4223**	96	9	0.5064	66
6	11	0.6321	64	10	0.5152	70	11	0.6200	38
8	15	0.8175	39	11	0.6214	71	12	0.7980	**28**
10	21	1.1675	44	12	0.7180	63	13	0.8618	29
12	22	1.2622	42	13	0.8173	57	16	0.9527	36
14	28	1.4911	39	14	0.8672	118	19	1.1124	32

The bold values represent the best values in both RMSE and processing time.

**Figure 7:**
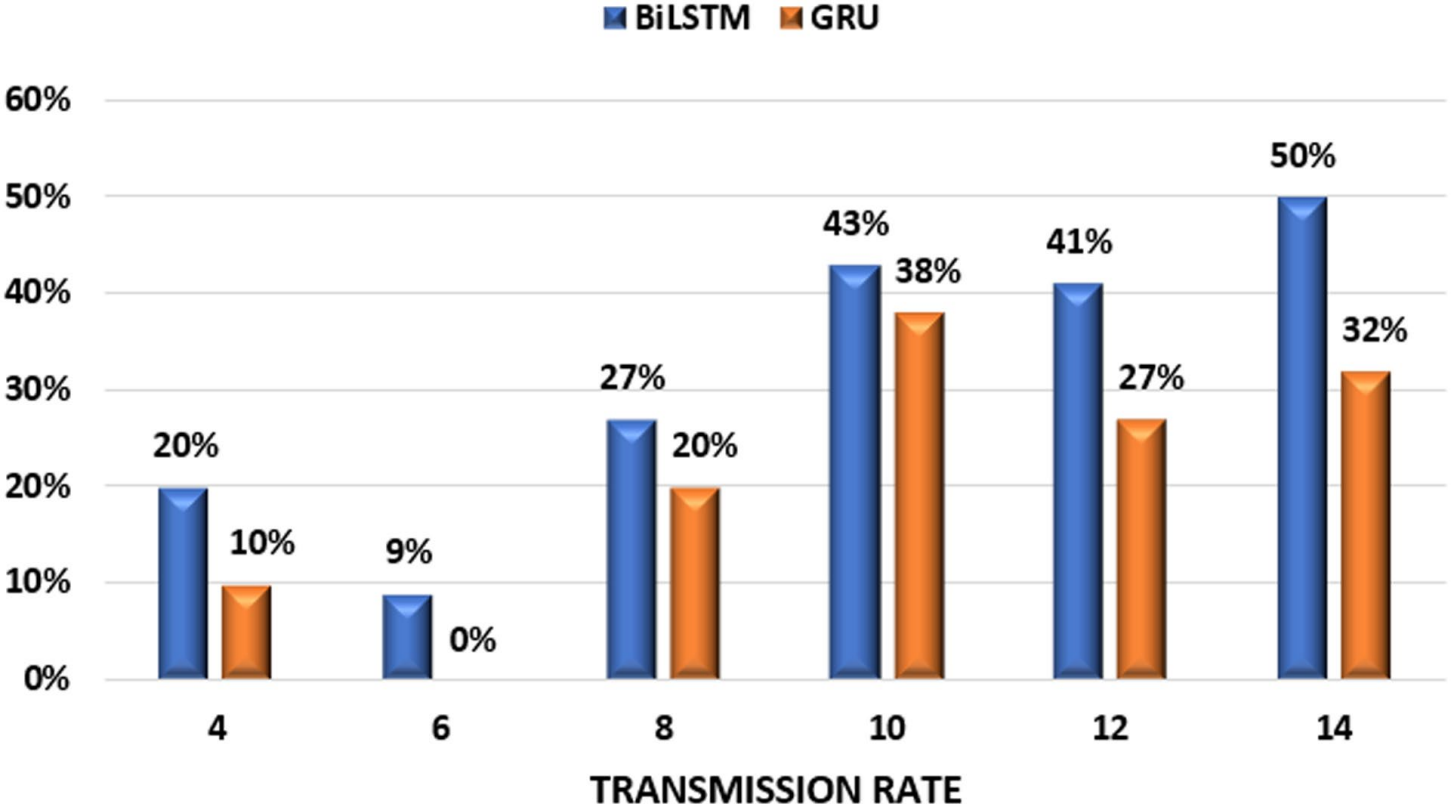
Maximum average improvement of DL predictors over LSTM for V2X traffic prediction.

Our research reveals a robust positive correlation between the complexity of DL architectures and their effectiveness in traffic prediction. As shown in Table 3, the proposed BiLSTM predictor consistently outperforms all baseline models (GRU, LSTM) across a wide range of transmission rates (4–14 packets/s). BiLSTM achieves significant performance improvement, ranging from 20 to 50% compared to the LSTM predictor and 11.1 to 26.3% over the GRU predictor. The most notable improvement is seen at a transmission rate of 14 packets/s, highlighting BiLSTM’s exceptional ability to handle high-traffic scenarios. While the GRU predictor performs comparably to BiLSTM in most cases, it experiences a substantial performance drop at 6 packets/s, mirroring the performance of the LSTM predictor. The LSTM model exhibits the lowest performance overall. Figure 7 visually compares the maximum average performance improvements achieved by the DL predictors.

Our experiments revealed a strong correlation between transmission rates and the performance of deep learning models (BiLSTM, GRU, and LSTM). All three models demonstrated peak performance at 4 packets/s transmission rate. At this rate, the BiLSTM model achieved the lowest RMSE of 0.4635 and MAPE of 8%, signifying a substantial 42.9% improvement compared to its performance at the lowest transmission rate. Similarly, GRU (RMSE: 0.5163, MAPE: 9%) and LSTM (RMSE: 0.5427, MAPE: 10%) also exhibited their best performance at this rate, with significant improvements of 52.6 and 64.3%, respectively.

**Figure 8:**
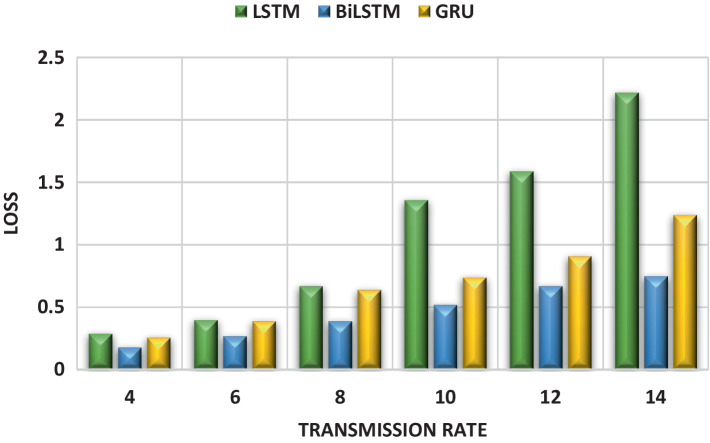
Training loss of DL models for different transmission rates.

Figure 8 complements this finding by illustrating the training loss for each DL model (BiLSTM, LSTM, and GRU) across different transmission rates. Lower training loss indicates better model performance. Consistent with Figure 6, the plots show the lowest loss at 4 packets/s for all models (0.18, 0.29, and 0.26 for BiLSTM, LSTM, and GRU, respectively). Conversely, the highest loss occurs at 14 packets/s (0.75, 2.22, and 1.24 for BiLSTM, LSTM, and GRU, respectively). Notably, the BiLSTM model consistently achieves the lowest training loss across all transmission rates, suggesting its potential superiority.

Network throughput, a measure of QoS, is also positively impacted by lower transmission rates. This aligns with our findings, as lower transmission rates allow for better predictions, potentially reducing network congestion and improving overall network efficiency.

**Figure 9:**
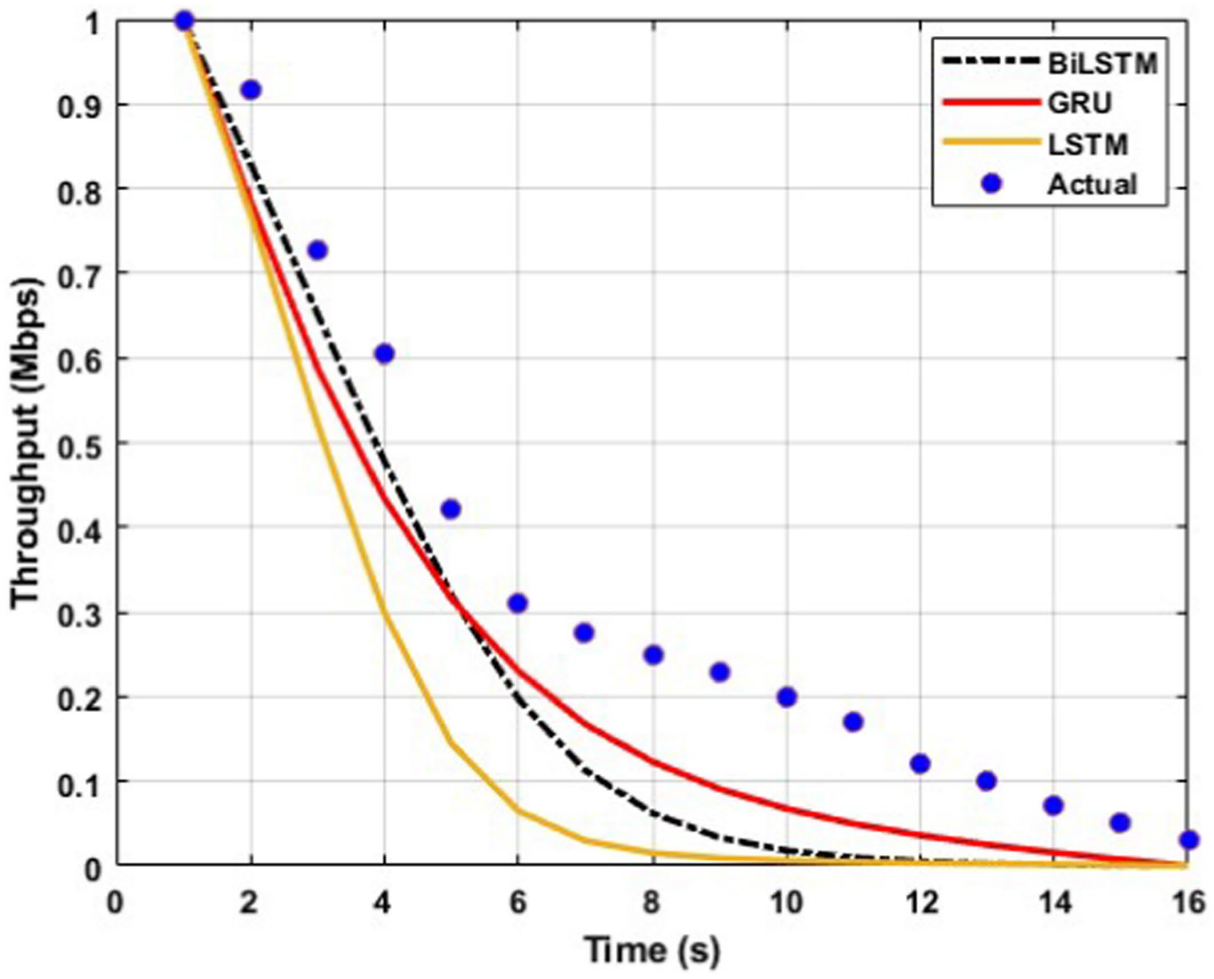
Predicted output for a transmission rate of 4 packets/s.

**Figure 10:**
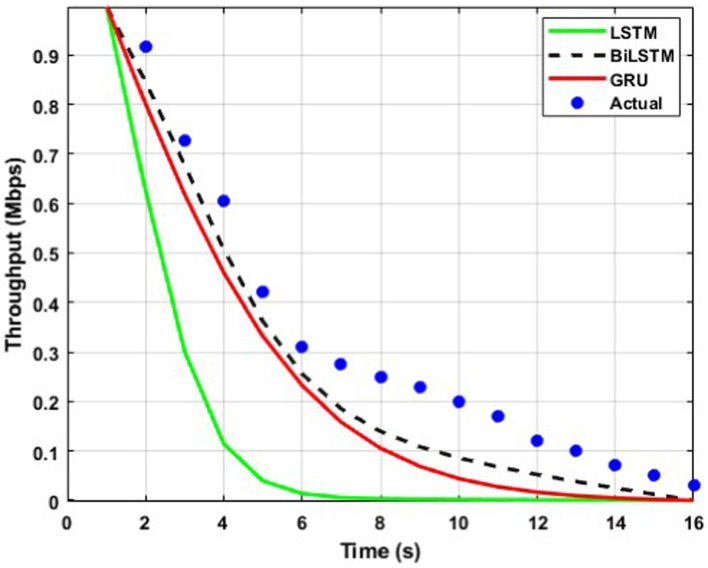
Predicted output for a transmission rate of 6 packets/s.

**Figure 11:**
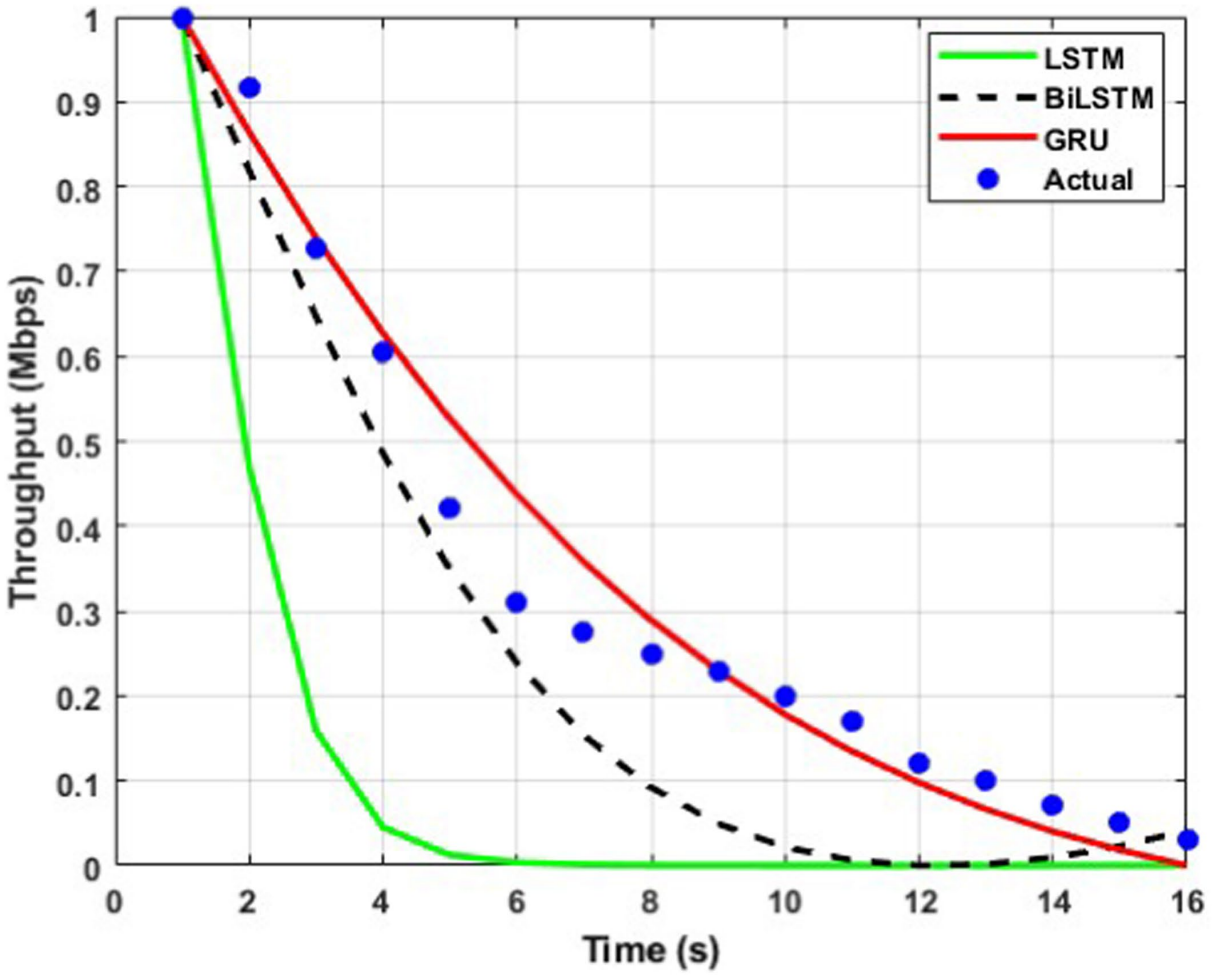
Predicted output for a transmission rate of 8 packets/s.

**Figure 12:**
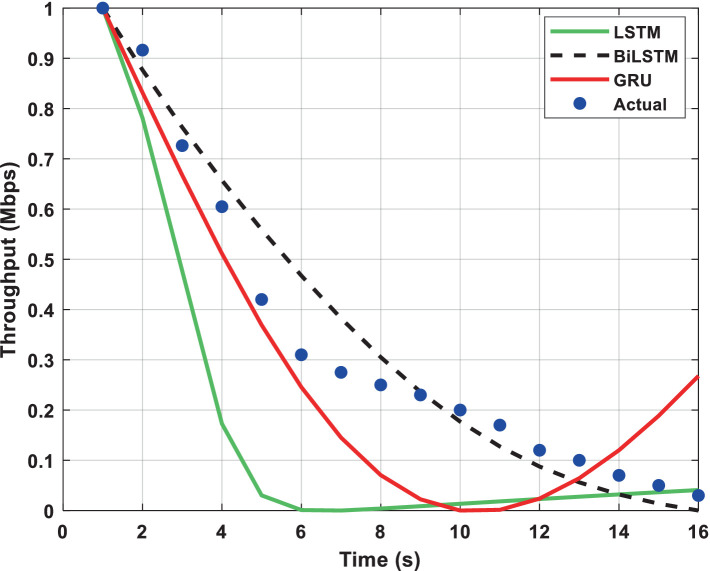
Predicted output for a transmission rate of 10 packets/s.

**Figure 13:**
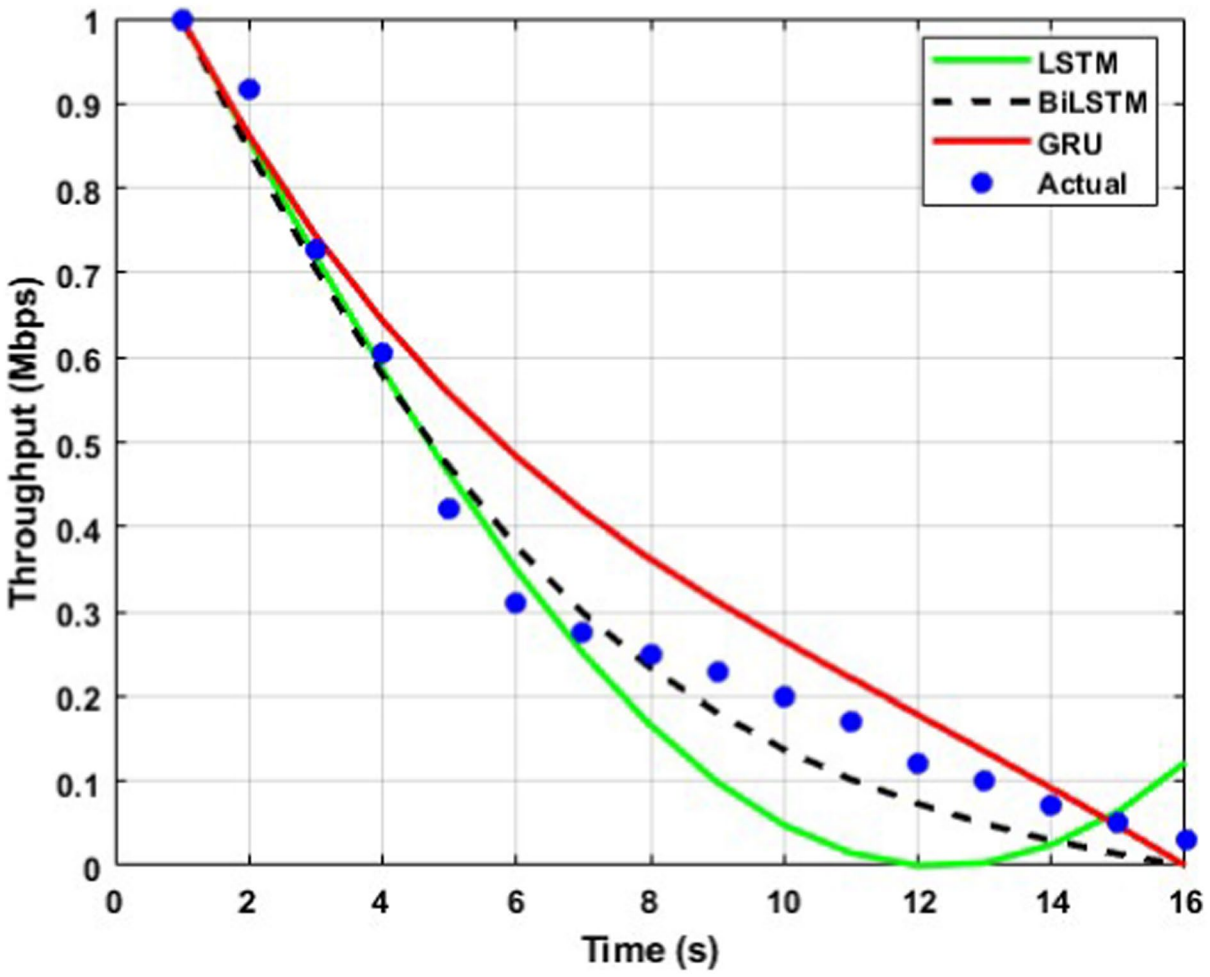
Predicted output for a transmission rate of 12 packets/s.

**Figure 14:**
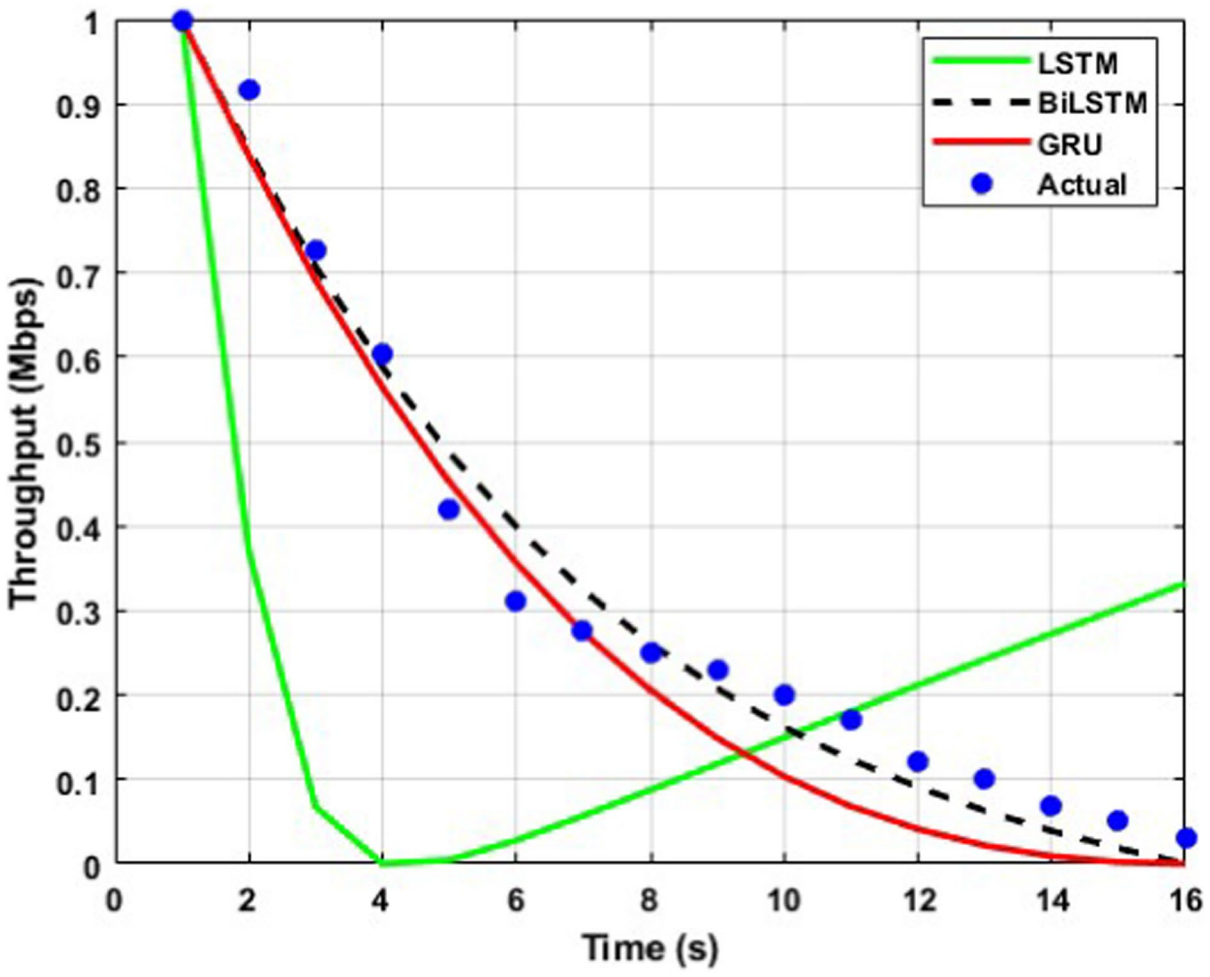
Predicted output for a transmission rate of 14 packets/s.

The throughput performance of three RNN architectures, namely BiLSTM, GRU, and LSTM, is comprehensively evaluated and compared to actual throughput values over time in Figures 9–14. A pervasive trend observed across all figures is a gradual decline in throughput, consistent with the inherent characteristics of the data analyzed.

A comparative analysis of the models reveals that BiLSTM consistently outperforms GRU and LSTM in predicting throughput, with its predictions closely approximating the actual values. This suggests that BiLSTM’s architectural design, which incorporates bidirectional connections to consider both past and future information, enables it to capture complex temporal dependencies within the throughput data effectively. The bidirectional connections in BiLSTM allow it to leverage both forward and backward information within the sequence, thereby enhancing its predictive accuracy.

GRU also exhibits satisfactory performance, closely tracking the actual throughput trajectory. This can be attributed to its simplified gating mechanism, which enables it to capture temporal dependencies while mitigating the risk of overfitting effectively. The reduced complexity of GRU’s architecture may also contribute to its improved generalizability and robustness compared to LSTM.

In contrast, LSTM displays inconsistent performance across datasets, with significant deviations from actual values observed in some instances, particularly toward the end of the time series. This variability suggests that LSTM may be sensitive to specific data characteristics or training conditions, which can lead to overfitting or challenges with vanishing/exploding gradients during training.

The comparative analysis of the three RNN architectures reveals that BiLSTMs and GRUs exhibit superior throughput prediction capabilities compared to LSTMs. BiLSTMs consistently demonstrate the highest accuracy, likely due to their ability to incorporate forward and backward information within the sequence. GRUs, with their simplified gating mechanism, may offer a compelling trade-off between predictive accuracy and generalizability, making them a viable alternative to BiLSTMs. The reduced complexity of GRU’s architecture may also mitigate overfitting and enhance model robustness compared to LSTMs.

## 6 Conclusion

In this paper, we investigated the effectiveness of various DL models for V2X traffic prediction. We proposed a novel approach that leverages RNNs for time series traffic flow forecasting. Our architecture explored the strengths of LSTMs in unidirectional and bidirectional (BiLSTM) configurations alongside GRUs to capture the inherent temporal dependencies in traffic data.

Our findings demonstrate the promise of the proposed approach, with the BiLSTM model achieving superior prediction accuracy compared to its counterparts. BiLSTM and GRU significantly outperformed the baseline LSTM model, highlighting the benefits of incorporating specific architectures for traffic prediction tasks. This work paves the way for developing more accurate and reliable V2X traffic prediction systems. These systems have the potential to significantly improve traffic management strategies, ultimately contributing to enhanced road safety and a more efficient transportation network.

Our findings also revealed a trade-off between prediction accuracy and processing time. The highest prediction performance was achieved at a transmission rate of 4 packets/s, while the lowest performance occurred at 14 packets/s. Interestingly, the GRU model exhibited the fastest processing time across all scenarios, while BiLSTM had the slowest. Similarly, the processing time for the LSTM and GRU models was the fastest at 8 packets/s. BiLSTM processing time was slowest at 14 packets/s despite being faster at other rates.

We plan to conduct a more comprehensive evaluation of the proposed DL-based predictors. This future work will explore the influence of various wireless network parameters, investigate the performance with different optimizers, and incorporate robust loss functions. We sequentially evaluate these factors to identify the optimal configuration for specific V2X communication applications. Further, to enhance traffic prediction accuracy, future research could investigate ensemble methods that leverage multiple RNNs or explore the influence of incorporating additional explanatory variables.

## Data Availability

The original contributions presented in the study are included in the article/supplementary material, further inquiries can be directed to the corresponding author.
